# Role of microbial electrosynthesis system in CO_2_ capture and conversion: a recent advancement toward cathode development

**DOI:** 10.3389/fmicb.2023.1192187

**Published:** 2023-07-14

**Authors:** Irwan Ibrahim, Mohd Nur Ikhmal Salehmin, Krishan Balachandran, Muhammad Farhan Hil Me, Kee Shyuan Loh, Mimi Hani Abu Bakar, Bor Chyan Jong, Swee Su Lim

**Affiliations:** ^1^Fuel Cell Institute, Universiti Kebangsaan Malaysia, Bangi, Malaysia; ^2^Institute of Sustainable Energy (ISE), Universiti Tenaga Nasional (UNITEN), Putrajaya Campus, Kajang, Malaysia; ^3^Agrotechnology and Bioscience Division, Malaysian Nuclear Agency, Kajang, Malaysia

**Keywords:** microbial electrosynthesis, CO_2_ sequestration, cathode modification, carbon capture, advanced material, biocatalyst, techno-economic, CO_2_RR

## Abstract

Microbial electrosynthesis (MES) is an emerging electrochemical technology currently being researched as a CO_2_ sequestration method to address climate change. MES can convert CO_2_ from pollution or waste materials into various carbon compounds with low energy requirements using electrogenic microbes as biocatalysts. However, the critical component in this technology, the cathode, still needs to perform more effectively than other conventional CO_2_ reduction methods because of poor selectivity, complex metabolism pathways of microbes, and high material cost. These characteristics lead to the weak interactions of microbes and cathode electrocatalytic activities. These approaches range from cathode modification using conventional engineering approaches to new fabrication methods. Aside from cathode development, the operating procedure also plays a critical function and strategy to optimize electrosynthesis production in reducing operating costs, such as hybridization and integration of MES. If this technology could be realized, it would offer a new way to utilize excess CO_2_ from industries and generate profitable commodities in the future to replace fossil fuel-derived products. In recent years, several potential approaches have been tested and studied to boost the capabilities of CO_2_-reducing bio-cathodes regarding surface morphology, current density, and biocompatibility, which would be further elaborated. This compilation aims to showcase that the achievements of MES have significantly improved and the future direction this is going with some recommendations.

**Highlights**

– MES approach in carbon sequestration using the biotic component.
– The role of microbes as biocatalysts in MES and their metabolic pathways are discussed.
– Methods and materials used to modify biocathode for enhancing CO_2_ reduction are presented.

## Graphical Abstract

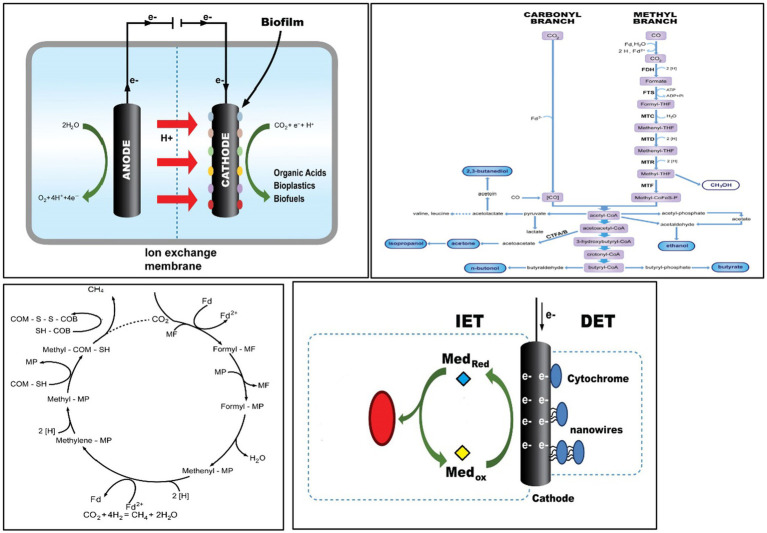

## 1. Introduction

The anthropogenic carbon dioxide (CO_2_) emission is responsible for global warming and climate. Furthermore, even with the implementation of the Paris Agreement at 33.5 Gt in 2018, climate change has increased to unprecedented levels in recent years. On the positive side, 2019 saw stable CO_2_ emissions at 33 Gt due to the implementation of renewable energy in the power sector (IEA, 2020). Then, in 2020, CO_2_ emissions in the energy sector dropped by around 5.2% (IEA, 2020) due to the lockdown caused by Covid-19. The slight drop in CO_2_ emission around the globe was an excellent short-term effect caused by the outbreak. As the efforts to recover from the pandemic, 2022 saw global CO_2_ emissions and energy rebound to reach their highest-ever annual level at a 0.9% increase, translating to 321 Mt. (IEA, 2023). Since 2020, the emissions have increased to 36.3 Gt with an average global CO_2_ growth rate of 3.0% annually from the early 2000s before decreasing to 1.2% annually for the last decade (2010–2019) (Mofijur et al., 2021). Thus, an effective and reliable method for capturing and storing CO_2_ capture and storage is vital to reduce and limit greenhouse gasses, especially CO_2_, as it is the highest emitted pollutant worldwide.

Carbon capture, utilization, and storage (CCUS) are one of the proposed ideas and methods used to address the problem and reach net-zero carbon emissions. CCUS is a holistic approach because it can be used in various sectors, such as manufacturing, construction, and oil and gas. As of last year, around 35 commercial facilities are applying CCUS to industrial processes, fuel transformation, and power, with a total annual capture capacity of almost 45 Mt. CO_2_ annually, exist around the globe, with 200 more yet to be completed in 2030, with 200 Mt. sequestration capacity (IEA, 2022). This method revolves around two categories, one of which is carbon capture and storage (CCS), in which the CO_2_ is collected, transported, and can be permanently stored in a facility to prevent it from ever reaching the atmosphere. One notable example is the Carbfix project in Iceland, where CO_2_ and hydrogen sulfide from the Hellisheidi geothermal power plant is injected into geothermal reservoirs, specifically porous basalt rocks, to form stable minerals for safekeeping (Sigfússon et al., 2018).

Another category is called carbon capture and utilization (CCU), where the CO_2_ generated by industry or recaptured from the atmosphere is used to produce carbon-based products such as fuel and chemicals. This method is used in enhanced oil recovery (EOR), where CO_2_ is injected into oil reservoirs to extract and displace remaining valuable gas where it is difficult to reach. This CCU method is applied worldwide, such as in Jilin Oilfield in China (Ren et al., 2016) and The Gorgon Project in Australia (Flett et al., 2009). This method could recover up to 470 million barrels of oil and 10–35% of the original gas in reservoirs (Hamza et al., 2021). Another application that has caught up with many researchers worldwide, which will be highlighted in this review, is the application of the microbial electrosynthesis system (MES) in CCU and how it will benefit scientists in addressing climate change generally and CO_2_ emission, specifically.

In an economic sense, the approach is favorable because the value-added product can be used or sold to a wide range of consumers, which could offset the cost of production itself instead of being stored in a facility. In other words, side profit can be generated by integrating these technologies in existing CO_2_-producing facilities, such as metal, power, and cement industries. Thus, the initiatives are incentivized to implement the bio-recycling system widely, contributing to the circular bioeconomy of MES, where the waste generated is recycled and converted to profitable products (Bian et al., 2020). Also, the design and scalability of MES can be easily implemented using existing fuel cell technology as both essentially run on the same principle, design, and component. For example, a small cluster of simple, small-size MES stack in hybrid series and parallel configuration can be used as the scaling-up strategy in implementing this system with a projected surface/volume ratio of 1 cm^2^/mL using flat, multi-chamber reactors (Jourdin et al., 2020).

MES is a bio-electrochemical system (BES) that harnesses and reduces CO_2_ into other beneficial carbon compounds using biocatalysts. This method combines existing CCU technologies and incorporates biotic components in the system that harness CO_2_ as a substrate and transform it into value-added products. However, up to this day, the commercialization of the MES system is far from reality because of some technical barriers that need to be solved to scale up system feasibility. These issues include the energy-intensive requirement of downstream processing and anode material cost, poor selectivity of the valuable product, and the interaction of cathode catalyst and biocatalyst, the most crucial aspect in determining its practicality and efficiency because it is the core part of MES. Thus, this review will concentrate on the recent progress of cathode catalyst fabrication from the past decade by measuring and comparing its performance from various angles in MES.

## 2. Fundamentals of CO_2_RR through MES

Biotic CO_2_ reduction reaction (CO_2_RR) could offer a broader range of possibilities than conventional methods in terms of economic feasibility given its self-generating capability, the low cost of microbes, and its low energy consumption in terms of temperature and pressure as it could operate in mesophilic conditions (Dessì et al., 2021). To this day, the most well-known method of CO_2_RR is photosynthesis. This process captures and converts CO_2_ from the atmosphere and is used for growth and energy storage in the form of carbohydrates. However, CO_2_ fixation using microbes could have a significant impact because it could be independent of sunlight, had a faster rate of reaction or growth, and was easy to manipulate. These processes’ derivations have many applications, such as in foods, feeds, fuels, and chemicals such as biogas (He et al., 2023), alcohol (Izadi et al., 2021), organic acids (Im et al., 2022), and bioplastic (Zhang et al., 2021).

**Figure 1:**
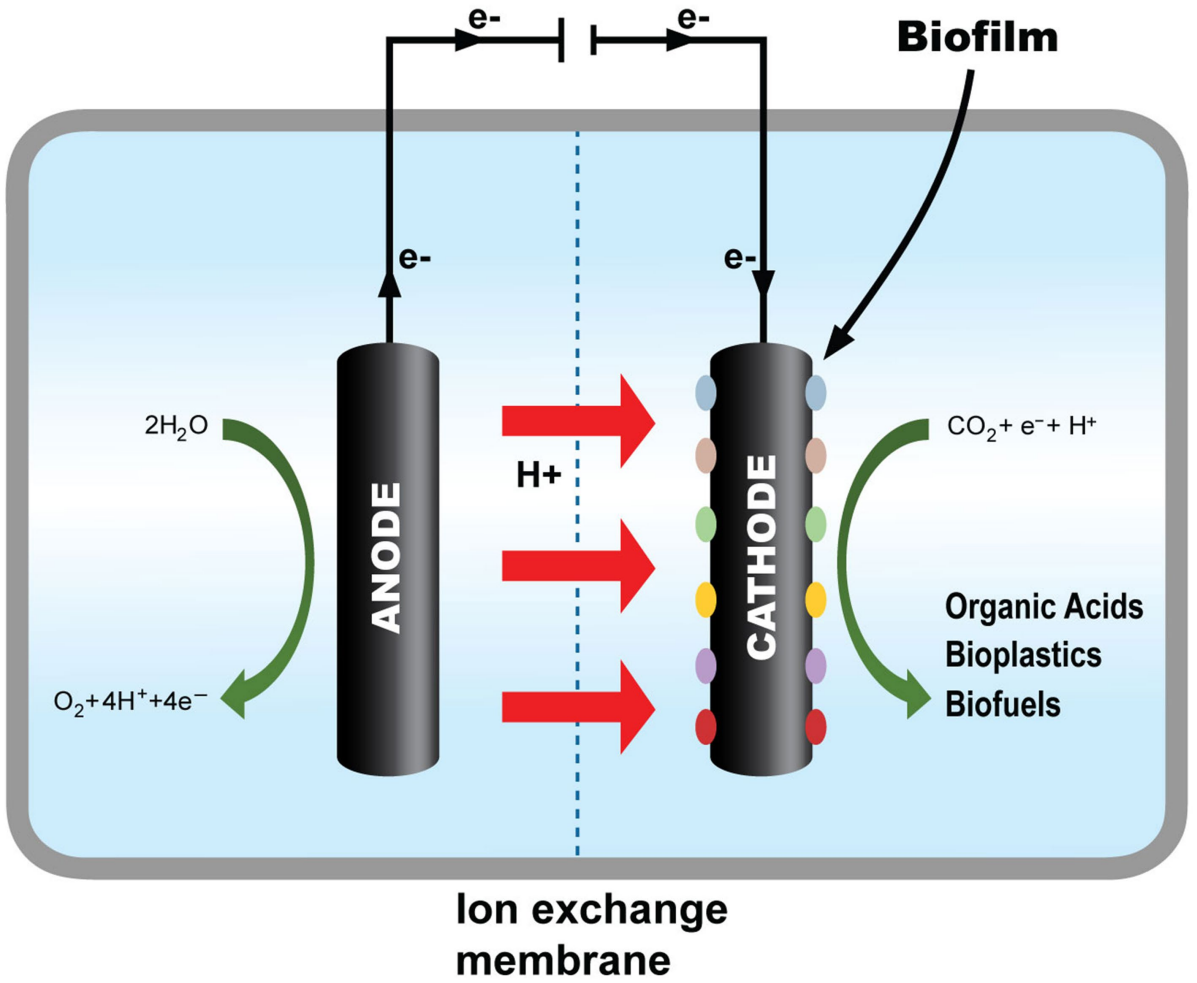
Fundamental illustration of MES.

In applying this process, several microbes were studied, ranging from archaea, bacteria, eukarya, and algae in biochemical CO_2_RR. Moreover, the biological pathways of these mechanisms were studied, and a few possible routes are widely suggested. The biochemical method of CO_2_RR, in general, is more favorable than conventional chemical methods because it uses less or no heavy metal and transition metal as a catalyst, which can lead to serious adverse effects on human health and the environment. In addition, this synthesis method can produce a more diverse range of products by manipulating different microbes and operating environments in the system because the microbes react differently according to the environment. This method could also treat municipal and industrial wastewater and recover the contained energy as hydrocarbon products, the leading factor in CO_2_ emission worldwide (Lu et al., 2018) (Figure 1).

**Figure 2:**
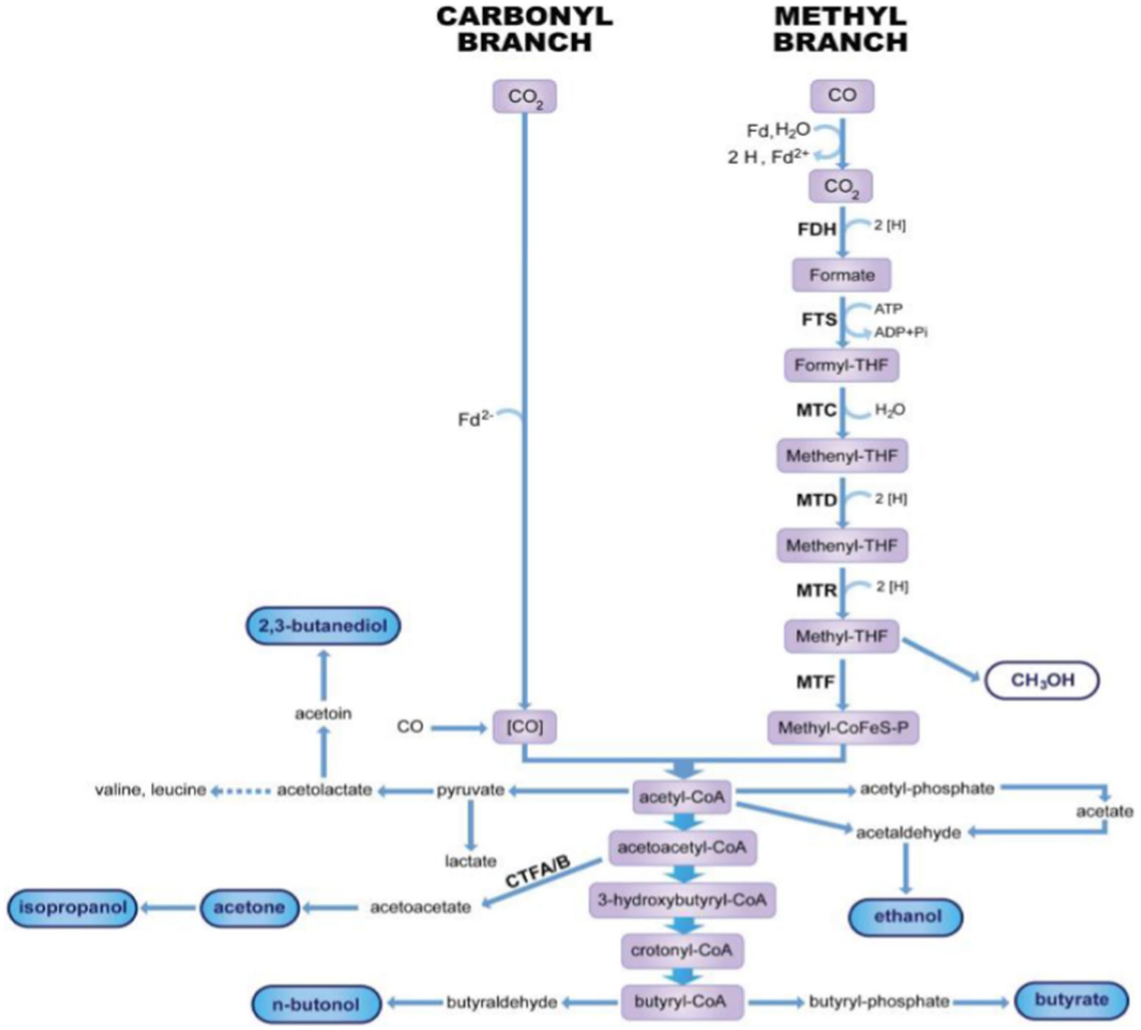
Wood-Ljundahl pathway of CO_2_RR in electrogenic microbes.

The electrogenic microbes also metabolize substrate differently depending on the species. In bacteria, acetogens such as *C. aceticum* and methanogens such as *M. maripaludis* undergo CO_2_RR through the Wood-Ljundahl pathway (WLP), which reduces CO_2_ into formate to acetyl Co-A before it is further synthesized to alcohol, acetate, and butyrate (Ragsdale and Pierce, 2008). WLP are two linear branch pathways, methyl and carbonyl branches, in which two CO_2_ molecules are converted to one molecule of acetyl Co-A. The methyl branch pathway involves CO_2_ being reduced to formic acid before it is bound in an ATP-dependent reaction to the tetrahydrofolic acid (THF) cofactor. Then, water is removed and reduced to methyl intermediate before it is transferred to another cofactor called the corrinoid iron-sulfur protein (CoFeS-P) to prepare the methyl molecule for condensation reaction. In the carbonyl pathway, the CO_2_ is reduced to carbon monoxide, which is enzyme-bound CO dehydrogenase (CODH) and acetyl Co-A synthase (ACS). The two pathways’ product is then condensed to form acetyl Co-A, the precursor to many organic products of MES that can be materialized through subsequent pathways, as in Figure 2. One such example is the formation of acetate, where acetyl Co-A is converted to acetyl-phosphate with the help of phosphotransacetylase (PTA) before acetate synthesis together with ATP via acetate kinase (ACK). Furthermore, acetogens are known to directly synthesize acetate by reducing CO_2_ with hydrogen and generate only 1.5 mol of ATP per mole of acetate produced, which is considered the thermodynamic edge of life (Poehlein et al., 2012). This explains why these autotrophs live in anaerobic environments without oxygen and are considered one of the earliest living organisms.

**Figure 3:**
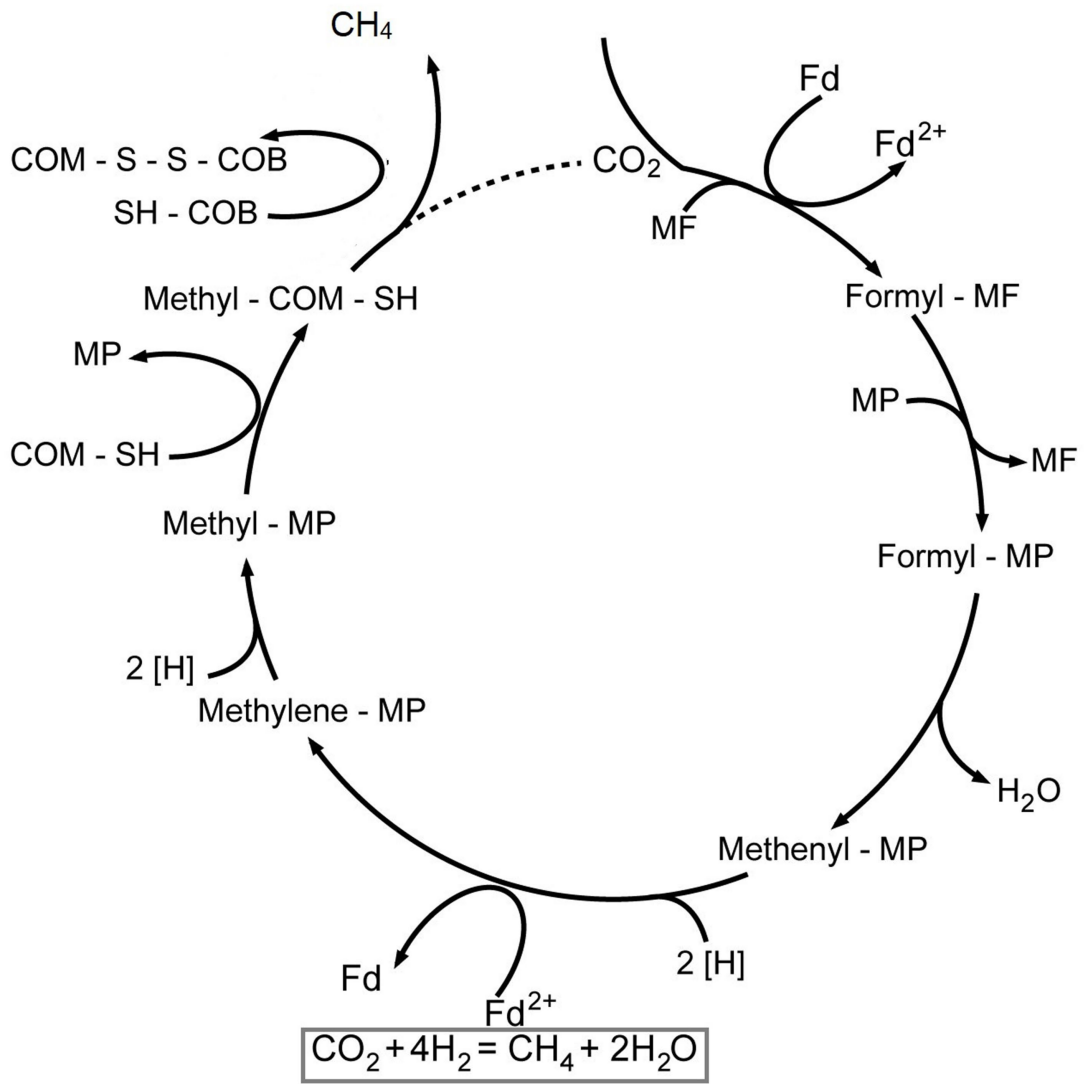
Wolfe’s cycle in methanogenic microbes that produce methane as a bioproduct.

Another metabolism harnesses in MES is methanogenesis, which anaerobic methanogens use to synthesize methane gas from CO_2_. This CO_2_RR starts by reducing CO_2_ into formyl by formyl-MF dehydrogenase containing methanofuran (MF). The second step involves transformation into formyl methanopterin (Formyl-MP) form, where it is further dehydrated and reduced into methenyl (Methenyl-MP), methylene levels (Methylene-MP), and finally to methyl form (MP-Methyl), respectively. Then the methyl group is then transferred to an enzyme called coenzyme methane (CoM) and coenzyme B (CoB) to be reduced by methyl reductase to form methane which is illustrated in Figure 3. Unlike Wood-Ljundahl, which has a straight pathway, this process occurs in a cyclic pattern named Wolfe’s cycle, where energy conservation is achieved.

**Figure 4:**
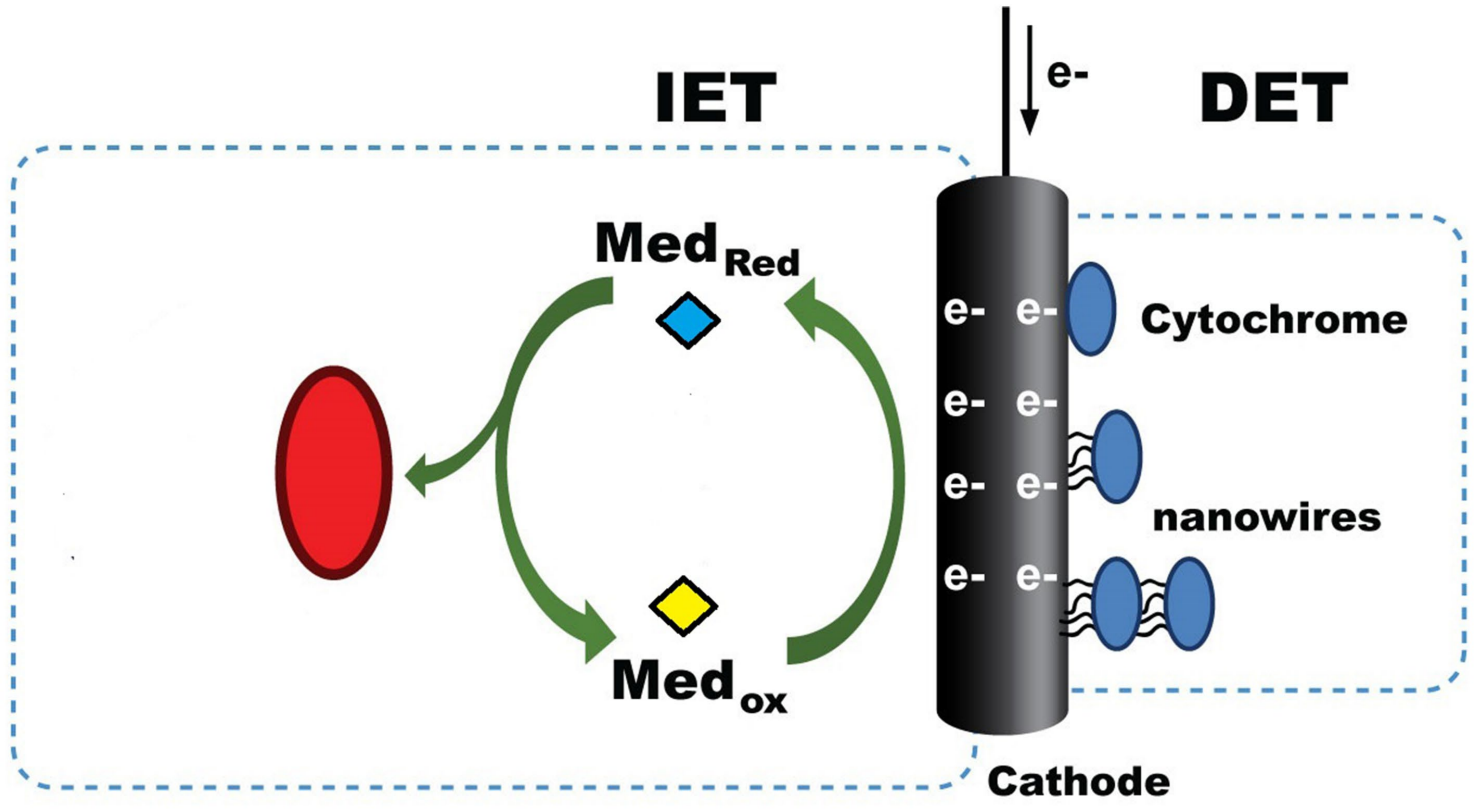
Electron transport mechanisms in MES.

Another important mechanism in CO_2_RR is the electron transfer process which is called extracellular electron transport (EET), in which the microbes use to drive the electrosynthesis; it is widely known that two main categories include under CO_2_RR, which are direct electron transfer (DET) and indirect electron transfer (IET) (Song et al., 2022). The DET process is the most straightforward and understandable principle in understanding how these electroautotrophs function in CO_2_RR. The microbes directly attached to the surface of the cathode will gain the electrons directly through the conductive outer membrane, such as cytochromes or nanowires, as in Figure 4 (Katsyv and Müller, 2020). In IET, the mechanism of how the microbes uptake electrons is much more complex than in DET because of the presence of intermediates. Microbes that suspend freely in the catholyte use IET to facilitate CO_2_RR mainly because the source of electrons is not directly accessible. This phenomenon occurs when the media contain compounds that are mediators and act as electron shuttles between the cathode and microbes, such as flavin, resazurin, hydrogen, and formate, to name a few (Vassilev et al., 2022). It is also suggested that IET involves direct interspecies called direct interspecies electron transfer (DIET), in which the nanowires are connected symbiotically between different species or through conductive particles in the system (Logan, 2009). This interaction is known to increase the bioproduction rate, such as methane and power production. However, the extent of this interaction is not thoroughly studied.

Thus, through the discussed fundamentals, MES harnesses these electrogenic microbes’ properties to catalyze the CO_2_RR using low-energy electricity to supply enough electrons for CO_2_RR to higher-chain organic compounds. This process is similar to the conventional electrolytic cell, where the anode dissociates the water molecules into electrons and protons before traveling to the cathode, where the CO_2_ will associate with these components to form different compounds. The only difference is that the reduction process in the cathode will be carried out by the electrogenic microbes present in the chamber through several pathways mentioned before, as illustrated in Figure 1, instead of the abiotic cathode.

## 3. Cell and cathode configurations for CO_2_RR in MES

**Figure 5:**
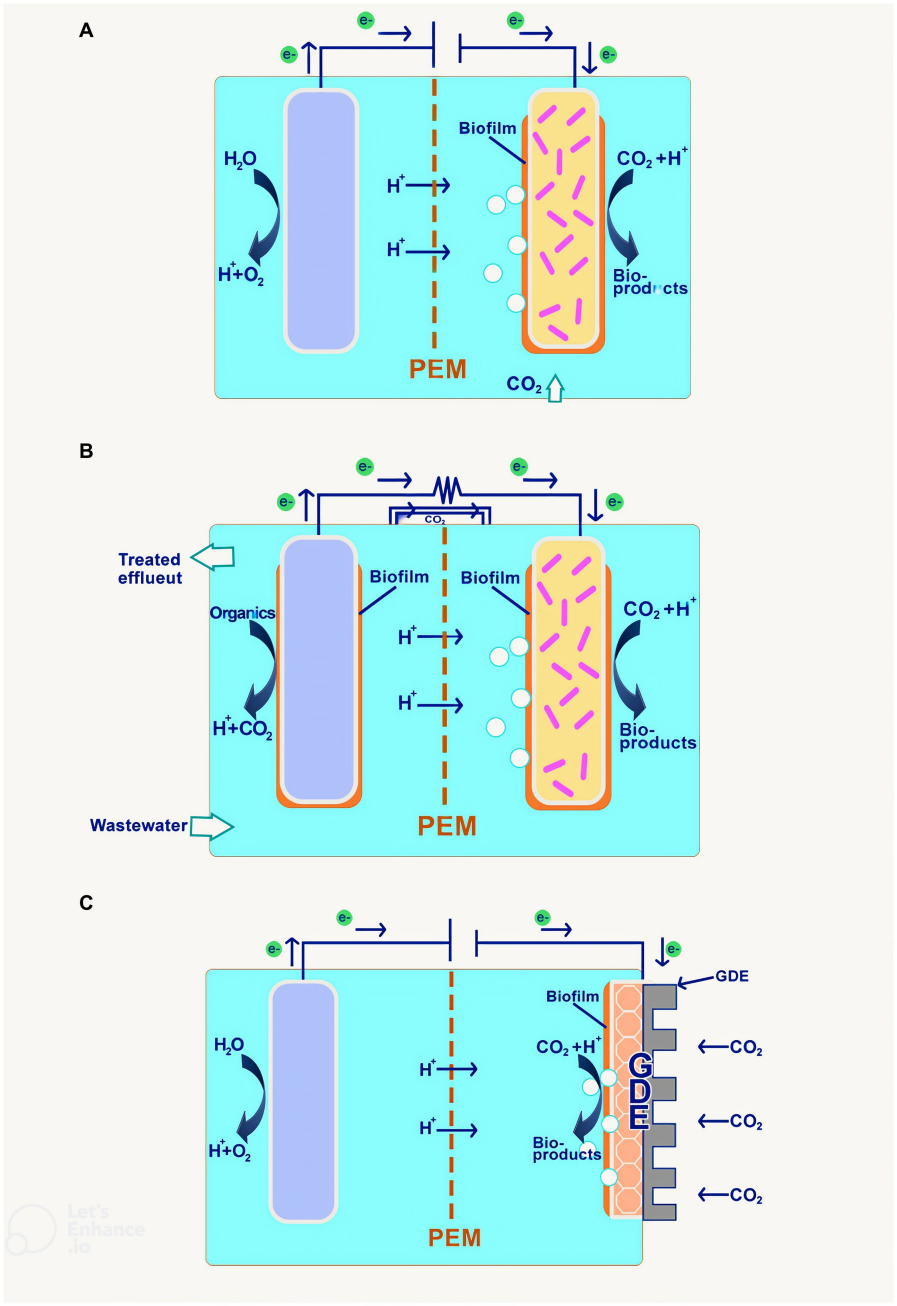
Proposed configurations of MES **(A)** Biocathode with abiotic anode; **(B)** Biocathode with bioanode; and **(C)** GDE biocathode.

MES system is an electrochemical system that harnesses the use of biotic components in the form of electrogenic microbes in its system to convert CO_2_ into value-added products through the use of low electrical current. The MES process can be performed using a microbial electrosynthesis cell, which has a configuration almost identical to an electrochemical cell except for biotic components on the cathode side. In this cell, the anode side is where the water is oxidized to produce oxygen and proton, which will then travel to the cathode via the ion exchange membrane. From there, the cathode catalyst, together with the electrogenic microbes on the cathode surface, called biofilm or suspends freely in the catholyte, reducing the CO_2_ present in the catholyte to produce a wide range of chemicals as illustrated in Figure 5A (Liu et al., 2023).

The second configuration uses the bioanode instead of the abiotic anode for oxidation. This is done by introducing anaerobic microbes in both the cathode and anode chamber, with different nutrient compositions in the media. This method could treat wastewater using oxidation to reduce the chemical oxygen demand (COD), producing current and biosensor simultaneously. Demonstration of the principle proves that COD removal is achievable by up to 87% and a maximum current of 0.369 A/m^2^ with an applied voltage of 1.4 (Xiang et al., 2017). The result indicates that as the voltage value proliferates, the amount of electrons correspondingly increases, leading to better biofilm growth and microbial activity. Furthermore, it could also be used to remove toxic contaminants such as perfluorooctanoic acid (PFOA) (Tahir et al., 2023) and sulfide (Kambara et al., 2023) through a similar configuration.

The cathode chamber in the MES system runs in anaerobic condition as the electrogen thrives in the condition; a CO_2_ sparger would be used to purge O_2_ out of the chamber while offering the environment with carbon-rich medium for the adsorption of the CO_2_ by the microbes and cathode for reduction processes. In addition, the carbon substrate for the process could be supplied in the form of bicarbonate ions derived from the CO_2_ capture via carbonation from industrial flue gas (Dessì et al., 2021; Roy et al., 2021). Also, a new approach has been used to introduce a gas diffusion electrode (GDE) as the mode of CO_2_ supply in the cathode chamber, as in Figure 5C. This method performs more efficiently in terms of CO_2_ mass transfer into the cathode and dissolution of CO_2_ in catholyte for biocathode, thereby increasing the system efficiency for electrosynthesis. An example of this can be explained through the comparative study of GDE-based MES and control, indicating that the carbon concentration in the media increase by almost five times in the GDE biocathode and product formation increases by almost double at the same flowrate (Rojas et al., 2021).

In many MESs, many experiments can be divided into two modes of microbial culture: single or mixed (Saratale et al., 2017). Most research was performed in single culture to study the microbes’ electron transfer mechanism or synthesize a specific compound. For example, an MES that consisted of pure culture of *Moorella thermoautotrophica* was analyzed to determine its electron uptake by manipulating the cell permeability of its cell structure (Chen et al., 2018). Mixed culture is the preferred method in studying the performance of MES by analyzing the compound synthesis. This method is more favorable because the condition and parameters of the system are less susceptible to environmental changes, contamination and are easier to control. These cultures can be found in wastewater sediments, ponds, lakes, and underground soil, generally consisting of *Geobacter* and *Schwanella* species.

## 4. Factors affecting cathodic performance

The biocathode, the core of MES, must be enriched at a particular stage before it can be utilized in MES. This phase, called microbial enrichment, is the first step where the intended microbes are cultured to multiply and attach to the cathode surface. In most cases, the microbes are taken from the anaerobic sludge of any wastewater treatment plant, where electrogenic microbes are usually present. The growth of microbes can be promoted by manipulating the potential applied to the system. Without this artificial control of potential, the anode potential in an MES fluctuates according to the load because of the redox potential of the electron carrier. Over time, as the MES operates continuously, the potential will drop until it approaches the thermodynamics of the substrate (Logan, 2009). Most research uses a potential lower than −0.6 V to achieve favorable electrosynthesis. Cathode potential is usually set at a much more negative value than the theoretical potential. A larger value of ΔE′^0^ (corresponding to a more negative cathode potential for a reducing reaction) can provide more energy for the growth of microbial cells. Meanwhile, a more considerable value of ΔE′^0^ also indicates a more considerable driving and a faster reaction rate for electrochemical reaction (Li et al., 2020).

Product selectivity is essential when deciding whether to choose pure or mixed culture. Despite its high productivity and selectivity, pure culture is significantly challenging to maintain because contamination and aseptic conditions play a more crucial role than mixed culture. However, previous studies confirm that product selectivity can be achieved at a higher rate even with diverse cultures using a chemical inhibitor that stops certain microbes’ growth. For example, a MES that specifically run to produce acetate comes from acetogenic types of microbes. However, in mixed culture, acetogen and methanogens can thrive simultaneously, generating unwanted byproducts and thus reducing yield. Sodium 2-bromoethanesulfonate (BrES) is the best-known methanogenic inhibitor, and it is used in several applications and fundamental studies in which methanogenic inhibition is required. Considering that the inhibitor structure is similar to methyl-coenzyme M, it competes with this molecule in the methanogenic pathway, thus hindering methane formation. BrES also affects other microbes by changing the microbial community structures by stimulating acetate metabolism and acetogenesis. It is also proven that by using BrES, the suppression of methanogen can be achieved up to 300 days of MES operation with acetate accumulation up to 10 g/L (Bajracharya et al., 2017). This technique is done in a two-way step where anaerobic sludge first undergoes heat treatment to denature heat-intolerant methanogens; the second step involves the enrichment stage where the addition of BrES in growth culture together with CO_2_ and H_2_ to proliferate the acetogens in autotrophic conditions before it is transferred into MES.

CO_2_ substrate in the form of bicarbonate ions (HCO_3_^−^) is the primary precursor for the CO_2_RR in MES. Thus, the concentration of this ion also plays a significant role in maximizing the performance of MES. As the concentration of bicarbonate ions increase, the yield of the desired product falls accordingly due to the inhibition effect (Mohanakrishna et al., 2018). Bicarbonate ions act as a carbon source for acetogenic microbes to synthesize bioproducts while also serving as buffers because basic ions counter-act the acid, thus maintaining the pH. Generally, a concentration of 2.5 g/L of HCO_3_^−^could be used to operate MES, although it can go up to 15 g/L. It should be noted that the higher the concentration, the productivity of products would decrease as well (Mohanakrishna et al., 2020).

The carbon chain elongation method is another method used to increase MES’s performance in producing higher-chain molecules compounds. This approach uses precursor carbon substrate added to the system to facilitate the chain elongation of CO_2_RR, such as formate, methanol, and acetate, to produce higher chain carbon compounds like butanol and butyrate. This method would reduce the metabolic pathway in Wood-Ljundahl in producing acetyl Co-A. It can be considered a shortcut, giving access to microbes to obtain more proton donors and use less energy for CO_2_RR. This approach is suitable for mixed culture-based MES as different microbes work synergically, producing products that are subsequently used as reactants for chain elongation.

Most MES systems perform well in mesophilic as electrogenic microbes thrive in that temperature range (Yang et al., 2021) and are usually done through temperature-regulated heaters. As the temperature increases, the growth of microbes, such as methanogen, is suppressed, leading to an increase in the acetogenic microbes’ distribution. However, a lower temperature would reduce the activities of the microbes, which leads to the lack of efficacy of MES. Certain thermophilic microbes can perform CO_2_RR, which explains why the heat treatment eliminates methanogens in MES. Microbes such as *Moorella thermoautotrophica* are one of the studied organisms that can tolerate a higher degree of temperature than mesophilic organisms. It can produce acetate and formate at an accelerated rate of 23.2 and 2.8 fold at 55°C than 25°C through the immobilized cathode method with carbon nanoparticles (Yu et al., 2017). However, this organism is gram-positive microbes with a thick, less electroconductive peptidoglycan layer, which hinders electron transfer. Thus, to overcome this obstacle, chemical additives could be utilized to inhibit the production of this membrane. Chemical such as penicillin has been proven to reduce the peptidoglycan in *Moorella thermoautotrophica* by almost half (Chen et al., 2018); this is shown when penicillin at a concentration lower than 30 mg/L could increase cell permeability, which results in increasing formate and acetate at a rate of 1.96 and 2.23 higher against the control. This finding could be helpful in future studies using other gram-positive microbes in MES and thermophilic conditions.

## 5. Material selection for cathode development

### 5.1. Abiotic cathode

Generally, the cathode material for use in MESs should be able to accept electrons, have a high surface area to facilitate the CO_2_ or other small organic molecules, and accommodate biofilm growth. The pivotal success of MESs hangs upon their ability to accommodate biofilm growth, as has been discussed and upheld rigorously in past studies, as the presence of microbe is significant for MES to work (Zhang et al., 2013; Aryal et al., 2019b; Prévoteau et al., 2020). This account implies that the cathode in MES must be biotic and opens a question: can the cathode in MES be abiotic? The term “abiotic” means the absence of living microbes on the electrodes acting as catalysts. By excluding the need for accommodating microbial population on the cathode, in theory, it should open a wide variety of options for the types of material that can be employed as an abiotic cathode in MESs. According to Aryal et al. (2017), previous studies on the role of abiotic electrocatalysis in MESs resulted in abysmal findings. Simply put, CO_2_RR using abiotic electrochemistry was never identified. In turn, this finding solidifies that the bioelectrocatalysis of CO_2_RR is the main bulk of the process happening in MESs.

So, why are abiotic cathodes in-applicable in MESs? One reason is the abiotic electro-reduction of CO_2_, which requires a very high input current (above 1,000 A/m^2^) to work (Gabardo et al., 2019). As a result, the cathode becomes a hostile environment for the microbes to grow. According to Prévoteau et al. (2020), MSCs work at an average current density of 100 A/m^2^, much lower than abiotic CO_2_R technology. The conductivity of media used in MES is 20 times lower compared to another abiotic electrochemical system, such as an electrolyzer (Gilliam et al., 2007).

Furthermore, although the use of noble metal, such as platinum, as an electrode drastically improves the efficiency of MES, the high price and potential of being a toxicant prove to be a significant hurdle for further advancement of this technology (Khurram et al., 2023). The incompatibility between the condition required for the abiotic cathode to work and the requirement of a microbe-conducive environment of MES made it impossible to bind these two factors in one cell. Therefore, the best approach to this situation is to employ MES in a case only suitable to abiotic cathode MES.

### 5.2. Biocathode

#### 5.2.1. Carbon-based biocathode

Carbon-based electrode, the most widely used material in all types of fuel cell technologies, has been studied and applied in this technology for decades mainly because they can conduct electricity efficiently and it is abundant, chemical resistant, and cost-efficient to be synthesized. The carbon-based electrode also exhibits biocompatibility properties because it is a non-toxic material, thus harmless to the microbiome, and can be integrated easily in MES. Different types of carbon-based electrodes that have been used and studied for cathode materials are summarized in Table 1. Standard graphite plates and rods can be categorized as planar or 2D materials because their structure consists of carbon atoms arranged in Sp2 hybridized orbitals with two other atoms for three lobes in a flat trigonal arrangement. This creates a sheet with a hexagonal structure with one unhybridized P orbital left on each particle, and these orbital forms Pi (π) bonds that merge into an extensive network on their own. These sheets are held together with weak Van der Waals forces that explain the graphite’s weak mechanical strength.

**Table 1:** List of carbonaceous biocathodes.

Cathode materials	Inoculum	Applied voltage (mV vs. SHE)	Current density	Products	Yield	References
Graphite plate	*M. thermoautotrophica*	−356 mV	NR	Acetate	49.35 mmol/m^2^	(Chen et al., 2018)
				Formate	13.62 mmol/m^2^	
Graphite plate	Enriched chemolithoautotroph	−1,400 mV	−50,000 mA/m^2^	Acetate	1.8 g/L	(Roy et al., 2021)
	*C. ljungdahlii*				1.10 g/L	
Graphite rod	*M. maripaludis*	−700 mV	219.61 mA/m^2^	Methane	21.85 mmol	(Mayer et al., 2019)
Graphite block	*C. ljungdahlii*	−1,200 mV	NR	Acetate	5.56 mmol	(Im et al., 2022)
				Formate	6.01 mmol	
				Lactate	0.77 mmol	
Carbon cloth	Salt marsh sediment (SM)	−1,200 mV	−1,800 mA/m^2^	Methane	4.90 mmol (SM)	(Alqahtani et al., 2021)
					3.80 mmol (M)	
Carbon cloth	Mangrove sediment (M)	−1,000 mV	−1,500 mA/m^2^	Acetate	1.50 mmol (SM)	67
Carbon cloth	Anaerobic sludge (AD)	−680 mV	NR	Acetate	3.10 mmol (M)	69
Carbon felt	Anaerobic sludge (AD)	−880 mV	NR	Acetate	36.66 mmol/L	(Ameen et al., 2020)
Graphite felt	Activated sludge	−1,200 mV	NR	Butyrate	19.1 mmol/L	(Izadi et al., 2021)
				Butanol	6.80 mmol/L	
Carbon fiber brush	Anaerobic sludge	−900 mV	NR	Acetate	630 mg/L	(J. Zhang et al., 2022)

^*^NR, Not reported.

These 2D materials are some of the earlier examples of materials used in the MES as the cathode base for synthesizing bioproducts. Although essential in structure compared with new materials, it performs CO_2_RR well by acting as a base for microbial growth and current conductor. An example is the use of graphite plate for acetate synthesis via the direct utilization of CO_2_ from the brewery industry, demonstrating the viability of this material by producing acetate at a rate of 0.26 g/L.d (Roy et al., 2021). The experiment also compares pure culture *C.ljungdahlii* and enriched mixed culture, where pure culture only obtained 0.138 g/L.d, which is lower than diverse culture. Another research was performed to study the operating conditions of MES using another 2D material, a commercial-grade graphite block to facilitate hydrogen-mediated CO_2_RR into acetate using a pure culture of *C. ljungdahlii* (Im et al., 2022). These results indicate the proven capability of basic planar materials, although the effect is inferior to the more developed 3D and advanced cathode.

Much research uses 3D materials made of carbon cloth (CC) and carbon felt (*CF*) as base or substrate electrodes for modification. CC comprises carbon atoms bonded to form a long cylindrical chain thinner than human hair. The fiber is highly stiff with high tensile strength, strong, flexible, and light in addition to its beneficial carbon properties and is used in many processes and disciplines. The intrinsic properties of CC make it a suitable candidate for conductive, metal-free electrocatalyst or base for increasing the mass loading of the catalyst and reaction active area (Shi et al., 2020). This material has integrated CC into the MES cathode to synthesize acetate and methane in varied quantities based on the microbes used (Alqahtani et al., 2021). Hydrophobicity is also one of the main issues that need to be addressed when using this material because it will hinder microbial attachment on the surface. Thus, a surface modification is required to increase its hydrophilicity to counter electrogenic microbes’ negatively charged surface membrane.

Another material, *CF*, is made of synthetic resin or cellulose made via needle punching and heat treatment (Huong Le et al., 2017). Graphite felt (GF), the following form of *CF*, is made by the graphitization process using higher temperatures. Its composition is similar to a CC but in a different morphological structure because carbon fiber is interwoven in a bundle resembling fabric, while *CF* is dispersed randomly. *CF* materials exhibit higher porosity, specific surface area, permeability, electro-resistivity, and better mass transfer than *CF. CF* has been applied in MES, and the demonstrated experiment yielded acetate of 36.66 mmol/L when the system-applied voltage was −0.68 V under optimum parameters (Ameen et al., 2020). This finding shows the capabilities of *CF* in reducing CO_2_ into a functional product as a primary, cheap, and stable cathode material. However, as these commercial materials’ properties have been fully realized, most studies only used either 2D or 3D materials to experiment on other variables of MES, such as operating conditions, scale-up design, and microbial properties.

#### 5.2.2. Composite-based biocathode

**Figure 6:**
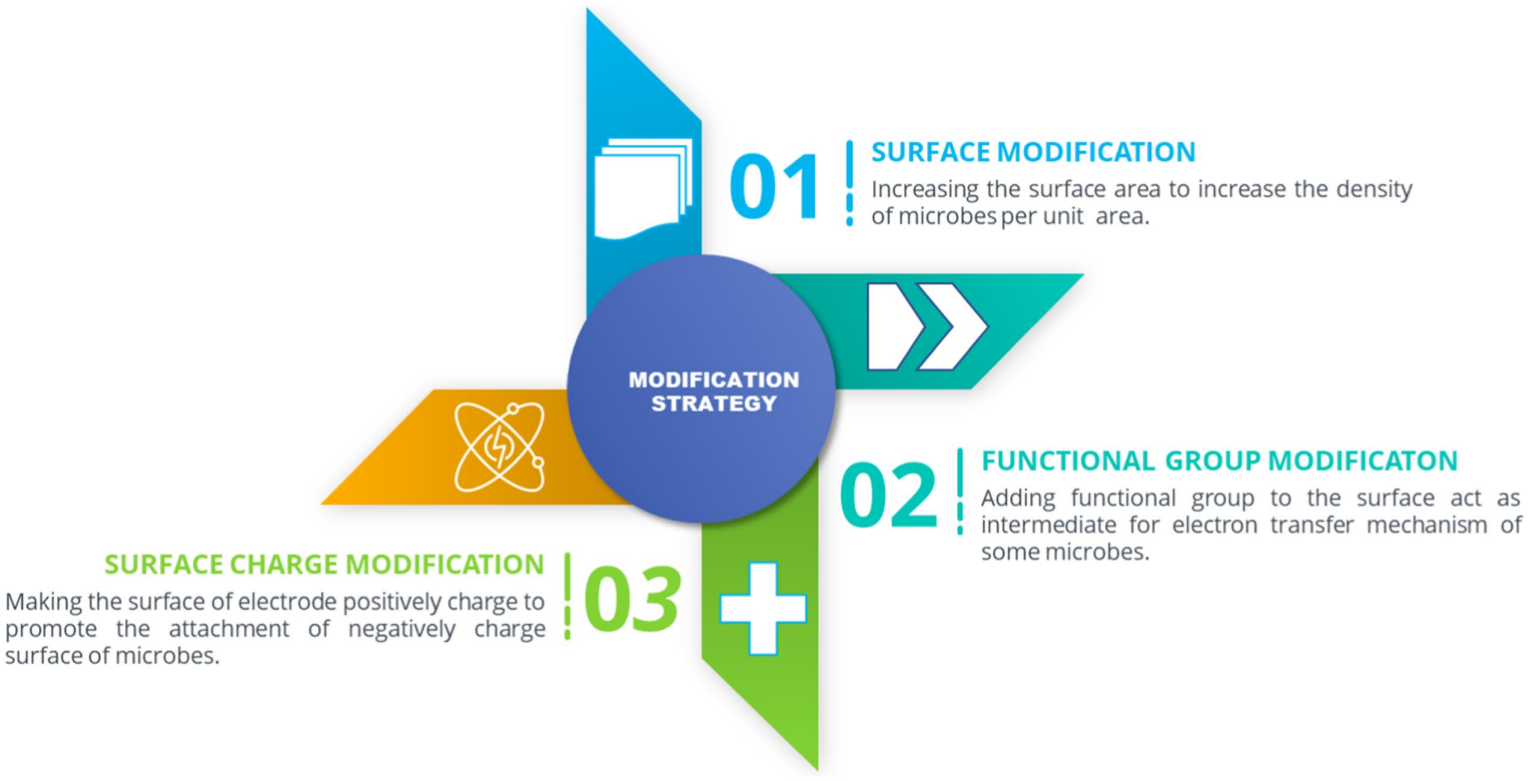
Modification approach of biocathode in MES.

Surface modification is another approach to effectively improve the physical and chemical properties. Thus, the biofilm attachment, biocompatibility, catalytic reaction, electron, and mass transfer could be improved. Surface modification has been categorized (Figure 6), including 3D modified surface, functional group attachment, and positively charged surface. 3D modified surfaces, such as carbon nanotube (CNT) and reticulated vitreous Carbon (RVC), outperform standard carbon electrodes regarding morphology and biocatalytic interaction with microbes. MES using RVC has demonstrated that it can achieve high microbial attachment and electron recovery to acetate up to 100% using electrophoretic deposition (Flexer and Jourdin, 2020). CNT also exhibits similar results by having lower internal resistance, stability, controllability, and uniform microporous structure up to 1 mm in diameter (Xie et al., 2012).

In recent years, newly discovered carbon structures are also used in MES to facilitate bio-electro reduction. This can be seen in using graphene as cathode material. Graphene, like other carbons such as graphite, is made entirely of carbon atom in a single layer in a hexagonal arrangement but is one atom thick and exhibit stronger hardness than steel. Graphene is also a flexible material because it can be used in liquid form and treated like carbon ink using a brush or spray for surface modification. Using this material, the performance of the MES has increased the production of acetate and butyrate by around 46%, and the current density increased by 85.7% (Hu et al., 2021). The *CF*-coated graphene biocathode exhibit higher surface-specific 3D surface area than plain and smooth *CF*, indicating that graphene carbon atoms arrangement can significantly increase biocathode biocompatibility.

Another almost similar materials, called graphene oxide (GO) and reduced graphene oxide (rGO), are also being tested to modify cathode catalysts. These two materials share similar structures of carbon arrangement but with different functional groups attached to the network, such as carboxylate, hydroxyl, and epoxy groups. GO material is made from the chemical exfoliation of graphite oxide, whereas rGO is made by the further reduction of GO to reduce the functional group of GO. To demonstrate the idea, rGO is used in an experiment using Manganese oxide/rGO hybrid biocathode (MnO_2_/rGO) to synthesize acetate and isobutyric acid (Thatikayala et al., 2021). The fabrication is done by mixing premade MnO_2_ and rGO with an oxidizing agent and heat treating to produce a precipitate. After the purification and drying, it is dissolved in nafion solution and airbrushed on CC. The MnO_2_/rGO biocathode outperforms the unmodified version by producing isobutyrate and acetate at a multiple of 2.09 and 2.19-fold higher, respectively. The formation of MnO_2_ nanowires and flowers’ crystalline nature, together with a hydroxyl functional group, enhances ionic diffusion and biofilm formation.

Furthermore, making the cathode positive makes the electrode surface prone to positive charges mainly because microbial cells consist of negatively charged surfaces. Therefore, to improve cell attachment and hydrophilicity, the cathode surface needs to be favorable to improve biofilm thickness. Several ways to do it are through treating the cathode with chemicals such as nitric acid, ammonia, and aniline. One study used nitric acid-treated granular graphite using a simple process where the standard granular graphite is pretreated with 5% nitric acid solution before washing and drying. This increases volumetric acetate production by 1.4 times with coulumbic efficiency of up to 65% compared with untreated granules (Shakeel et al., 2020) since the surface of granular graphite has become more electropositive; thus, the biocathode can accommodate higher negatively charged microbial membrane. However, the selectivity of the product is affected due to the absence of the buffer in the catholyte, and the system operates in batch mode. This caused the pH fluctuation as the acid would lower the pH and force the microbes to shift their metabolites to synthesize ethanol from CO_2_. Therefore, the operating conditions of MES should also be considered in optimizing the efficiency and selectivity of the bioproducts produced.

Another conventional method used in manipulating the cathode is via electrodeposition method. This method is mature enough to be applied in any application regarding electrochemistry. This method could increase the surface area, make the surface cathode more positive, or add functional group attachment, further improving MES’s electrocatalytic activity. One notable example is by using Tin, a well-known electrocatalyst for formation synthesis that is abundant and cost-efficient. The 4 × 4 × 0.5 cm Sn-based biocathode is prepared by electrodeposition on *CF*; the salt bath is made by mixing SnCl_2_ with citrate as a chelating agent in the 4 mA galvanostatic electrodeposition coating at three different durations (Qiu et al., 2023). A mediator in the formate is also added into the MES to drive the electrosynthesis process faster, resulting in acetate production of up to 0.32 g/L.d for the most extended deposition duration as in Table 2. This shows time plays a significant factor in the electrodeposition process as the accumulation of electroactive metal oxides proliferates against time. Another interesting approach in electrodeposition is observed in a study of simultaneous *in-situ* iron deposition with nickel and CO_2_RR process, which could produce various compounds from methane and acetate to alcohol, depending on the operating pH (Gomez Vidales et al., 2021). The metals are deposed by adding FeSO_4_ and NiSO_4_ salts into nutrient-rich catholyte after the MES has matured. NiSO_4_ addition caused the current to rise by 35% and the production of CH_4_ by 24%, while FeSO_4_ led to a current increase, reaching 370 mA. In contrast, CH_4_ production heightened to 0.73 L/d at an applied voltage of 2.8 V. Interestingly, after removing both salts in the media, the performance of MES still constant, meaning successful deposition of Ni and Fe. The observation of the pH also indicates that the methanogenic phase is prevalent at pH 7 to 8.5, while a higher pH will trigger the acetogenic phase of CO_2_RR.

**Table 2:** List of composite biocathodes.

Cathode materials	Inoculum	Applied voltage (mV vs. SHE)	Current density	Products	Yield	References
**Carbon felt**						
MnO_2_	Anaerobic sewage sludge	−1,350 mV	−3.70 mA/m^2^	Acetate	37.90 mmol/L	(Anwer et al., 2019)
Graphene	Mixed culture	−1,000 mV	2,600 mA/m^2^	Acetate	0.26 g/L	(Hu et al., 2021)
				Butyrate	0.09 g/L	
Fe_3_O_4_/GAC	Enriched mixed culture	−1,050 mV	40,300 mA/m^2^	Acetate	5.14 g/L	(Zhu et al., 2019)
TiO_2_	Anaerobic sludge	−900 mV	7,260 mA/m^2^	Acetate	8.86 g/L	(Das et al., 2021)
Rh			6,110 mA/m^2^		6.65 g/L	
Neutral red/Nafion	Anaerobic sludge	−1,200 mV	NR	Acetate	340 mmol/L	(S. Li et al., 2022)
Nano-titanium carburised electrode	Activated sludge	−1,000 mV	2.75 mA/m^2^	Acetate	234 mg/L	(Hu et al., 2021)
HNO_3_ treated Granular Graphite	Anaerobic sludge	−1,050 mV	−512 mA/m^2^	Acetate	4.40 g/L	(Shakeel et al., 2020)
**Graphite felt**						
Stainless steel	*C. ljungdahlii*	−1,100 mV	−1,000 mA/m^2^	Acetate	10.50 mmol	(Bajracharya et al., 2015)
	*S. thermospinisporus*	−1,300 mV	20,000 mA/m^2^		9.33 mmol	
Graphite stick	*C. ljungdahlii*	−1,100 mV	−7,700 mA/m^2^	Acetate	10 g/L	(Bajracharya et al., 2017)
Molybdenum carbide	Proteobacteria	−850 mV	−5,200 mA/m^2^	Acetate	5.72 g/L	(Tian et al., 2019)
Graphene oxide-coated copper foam	*S. ovata*	−1,300 mV	−21,600 mA/m^2^	Acetate	1983.60 mmol/m^2^	(Aryal et al., 2019a)
Activated carbon GDE	Anaerobic digester effluent	−1,200 mV	−1,200 mA/m^2^	Acetate	1.30 g/ L	(Rojas et al., 2021)
MnO_2_/rGO	MES effluent	−830 mV	NR	Isobutyrate	15.90 mmol	(Thatikayala et al., 2021)
				Acetate	3.50 mmol	
Sn-modified electrode	Matured MES planktonic cells	−1,300 mV	−700 mA/m^2^	Acetate	5.38 g/ L	(Qiu et al., 2023)
*In-situ* deposition Ni-Fe	*Clostridium*	−1,500 mV	6.4 mA/cm^2^	Ethanol	8.00 g/L	(Gomez Vidales et al., 2021)
				Acetate	2.40 g/L	

^*^NR, Not reported.

## 6. Advanced cathode materials

Apart from the conventional electrode configuration discussed previously, several types of unconventional material or newly discovered methods of utilizing it in fuel cell applications, specifically in MES, are shown in Table 3. These approaches offer new ways of fabricating the cathode through more efficient and feasible mean possible. One example is the use of MXenes material, a newly discovered 2D material with high conductivity and surface area due to the multiple layers in its structure. Owing to these properties, the *CF*-coated titanium carbide MXene synthesized acetate, butyrate, and propionate at 1.6, 1.1, and 1.7-fold, respectively (Tahir et al., 2020).

**Table 3:** Some of the advanced biocathode used in MES.

Biocathode materials	Inoculum	Applied voltage (mV vs. SHE)	Current density	Products	Yield	References
3D-Bioprinted microbes	*S. ovata*	−1,000 mV	14,800 mA/m^2^	Acetate	58.40 mmol	(Krige et al., 2021)
Titanium carbide MXene carbon felt	Wastewater	−1,000 mV	−71.2 mA/m^2^	Acetate	0.77 g/L	(Tahir et al., 2020)
				Propionate	0.87 g/L	
				Butyrate	1.56 g/L	
Fluidized granular activated carbon	Effluent of MFC and AD	−1,250 mV	−4,080 mA/m^2^	Acetate	16 g/L	(Dong et al., 2018)
Reticulated vitreous carbon (RVC)	Enriched culture	−1,000 mV	−8,300 mA/m^2^	Acetate	8.2 g/m^2^	(LaBelle and May, 2017)

Another ingenious method of incorporating the existing engineering approach in MES is via the fluidized bed reactor principle. This approach offers many advantages compared with standard reactors, such as simple construction, excellent particle mixing, and large mass transfer with electrolyte substrate. A study indicates that the acetate production rate increased by 2.8 times through MES with 0.14 g/ L.d compared with the control using granular activated carbon (GAC) as the mobile cathode and the existing static *CF* cathode (Dong et al., 2018). This was achieved because the additional cathode has a massive surface area of 900 m^2^/g and an average pore diameter of 2.2 nm. In addition, further research modified the GAC with metal oxides such as Fe_3_O_4_, yielding acetate at a rate of 0.171 g/L.d which correlates to a 1.4 times better yield than an unmodified one (Zhu et al., 2019).

Furthermore, a new concept of bio-electrosynthesis is enzymatic electrosynthesis (EES). This method involves culturing microbes that contain specific enzymes to catalyze the reduction of CO_2_ and harvesting it in sufficient quantity before it is purified to be used on the cathode substrate surface. EES offers highly selective productivity without the unrelated metabolic pathways in the CO_2_ reduction process, which would generally increase MES’s substrate and energy consumption. For example, the heterodisulfide reductase supercomplex (Hdr-SC) of *M. maripaludis* is culture and extracted via centrifugation before it is purified through chromatography. The enzyme obtained is inserted into the cathode chamber without any chemical mediator, as typically used in MES. The result suggests the formate production of 3 μmol/h.cm^2^ obtained vs. the three times higher control (Lienemann et al., 2018). This technology, however, is still in its infancy as the cost of production is exponentially higher due to enzyme harvesting and purification steps.

Another futuristic approach used in MES is the synthesis of biofilm on the cathode surface using the 3D printer. This process involves mixing the electrogenic microbes with a biocompatible binder and injecting it directly onto the surface of the cathode substrate. This method has the potential for scalability as the technology has been used in different applications and cost optimization due to the relatively fast the cathode production process can be. An example is a study that used concentrated *S. ovata* strain and mixed it with alginate and cellulose-based hydrogel before printing it on the CC electrode. The result indicates acetate production at a rate of up to 0.68 g/L.d (Krige et al., 2021).

## 7. Coupling cathode with GDL for CO_2_RR

As discussed previously, the mass transfer of gaseous CO_2_ is a critical hindrance to improving the performance of the electroreduction of CO_2._ The solubility of CO_2_ in an aqueous electrolyte is 33 mmol at 298 K and 1 atm (Lucile et al., 2012). The amount of CO_2_ that can be dissolved in the solution is limited by the partial pressure of CO_2_, which is explained by Henry’s law. Lowering the temperature and increasing pressure can increase the amount of dissolved CO_2_ (Cook et al., 1990); however, these options are not practical for commercial applications and may not necessarily lead to higher current densities. So far, the primary solution to this problem has been the sparging method, which results in a significant loss of CO_2_ into the atmosphere (Mateos et al., 2019). GDEs are widely used to assist in transferring gas-phase reactants to the electrode surface where the electrochemical reaction occurs. Due to high CO_2_ mass transport and reduced diffusion lengths within the catalyst layer (CL), GDEs can achieve higher current densities than traditional electrodes (Durst et al., 2015). GDEs are commonly used in a range of electrochemical systems, including fuel cells (Ehelebe et al., 2022), electrolyzers (Mowbray et al., 2021), and sensors (Becker et al., 2023). In fuel cells, for example, the GDE acts as the electrode where the oxygen from the air and hydrogen from the fuel combine to produce water and electricity (Arslan et al., 2023). The GDE generates hydrogen and oxygen gas from water through the electrochemical reaction in electrolyzers.

**Figure 7:**
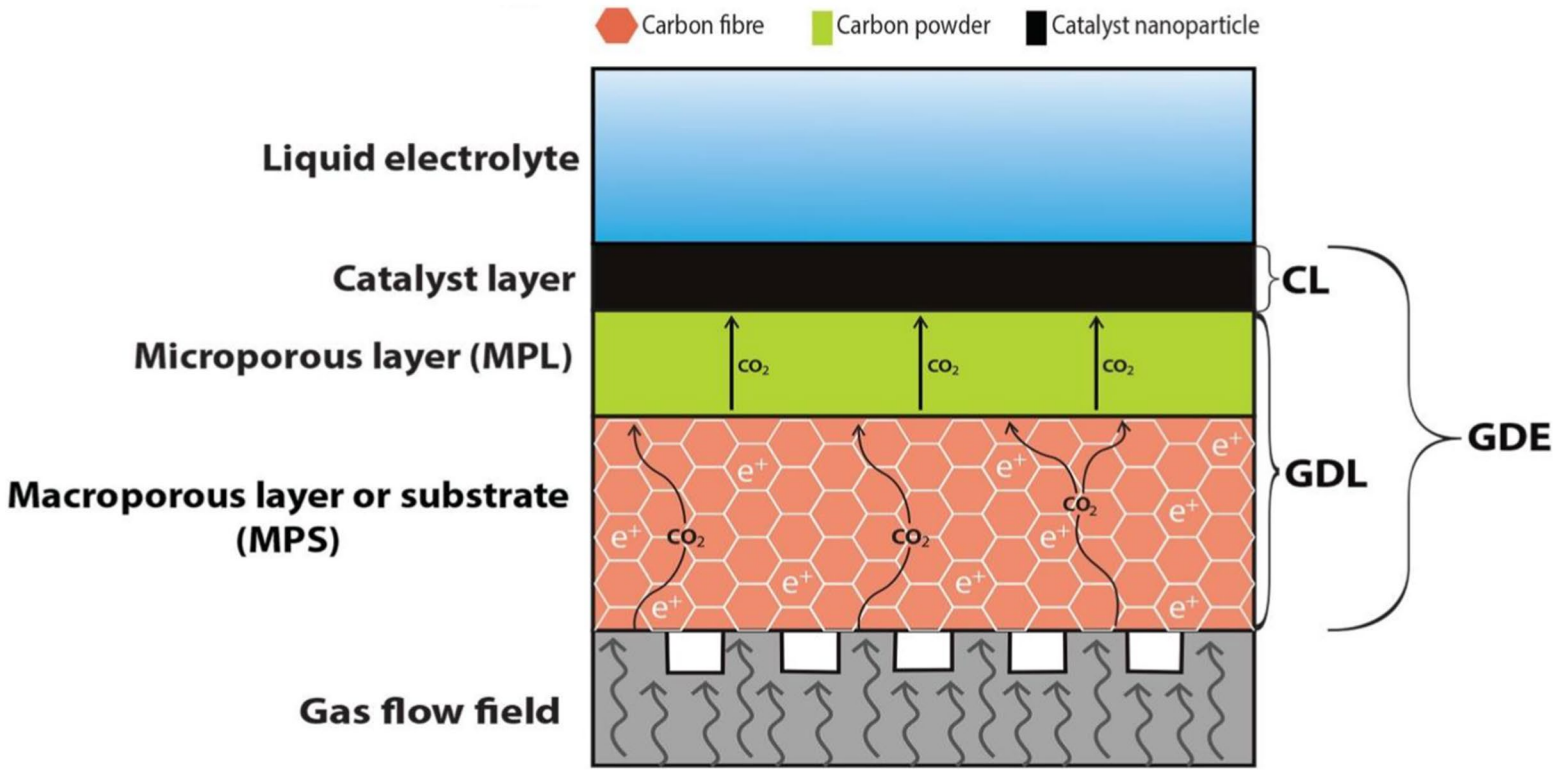
Cross-sectional view of GDE.

GDE is made up of a GDL with a CL on its surface. The gas diffusion layer (GDL), a porous layer between the CL and the gas flow channel or field, has two primary functions: it permits gas transfer toward the CL and provides mechanical support to the catalyst. The GDL is designed to be hydrophobic to prevent the electrolyte from clogging its pores to enable gas transport to the CL. GDLs have two primary categories: single-layer and dual-layer GDLs. A single-layer GDL comprises only a microporous layer (MPS), whereas a dual-layer GDL comprises an MPS and a microporous layer (MPL). Dual-layer GDLs are commonly used, especially in CO_2_ electrolysis, to prevent GDE flooding. A typical dual-layer GDL structure is shown in Figure 7, with the gas flow field directly contacting the MPS layer, which acts as a gas diffuser and current collector. The microporous layer is situated atop the MPS and typically consists of carbon and hydrophobic agents to regulate catholyte flooding.

The MPL is a thin material coated onto the electrode’s surface, typically made of carbon fibers or carbon black. The MPL provides a high surface area and a hydrophobic surface that allows the diffusion of gas through the electrode while preventing the infiltration of liquid electrolytes. On the contrary, the MPS is a more extensive and porous layer constructed from a conductive material, such as carbon paper or cloth. It acts as an MPL foundation and enables an electrically conductive pathway for the electrochemical reaction. Additionally, the MPS offers mechanical stability to the electrode and promotes uniform gas flow distribution throughout the electrode surface. The combination of MPL and MPS allows efficient gas diffusion and electrochemical reaction while preventing electrode flooding through the liquid electrolyte. Additionally, using a two-layer GDE can improve the selectivity and efficiency of the electrochemical reaction, as the MPL can be tailored to enhance the activity and selectivity of the electrocatalyst used in the reaction. Overall, GDEs with both MPL and MPS layers are a practical design for facilitating gas diffusion and electrochemical reactions in various applications, including the electroreduction of CO_2_.

The CL is responsible for creating an efficient triple-phase interface by forming microchannels that contain a thin layer of electrolyte, ensuring adequate gas delivery and contact between the electrolyte and catalyst (Han et al., 2018). For the catalyst to be effective, it must be covered by an electrolyte in equilibrium with the gas-phase reactant (Weng et al., 2018). Therefore, the wetting properties of the CL play a critical role in stabilizing gas concentrations at the interface and improving the interaction between the electrolyte and catalyst. Two main methods can be used for fabricating the CL: ink-based and ink-free. The performance and product distributions of the GDE are also affected by the amount of catalyst loading in the CL. Increasing the catalyst loading in the CL increases the current density by providing more catalytic active sites; however, this approach does not necessarily lead to better FE for the desired product (s) (Kopljar et al., 2014). The increased number of active catalytic sites can alter the GDE potential and, therefore, the product distribution of the electrocatalytic reactions. For example, during CO_2_RR, increasing Sn loading resulted in increased faradaic efficiency (FE) of CO and hydrogen evolution reaction (HER), leading to lower selectivity for the desired product, formate (Zheng et al., 2019). A higher catalyst loading typically results in a higher concentration of intermediates, altering the reaction pathways. For instance, increasing the Cu nanoparticle loading on a carbon-based GDL changed the product selectivity of CO_2_R to C_2_+ hydrocarbons and suppressed HER and C1 hydrocarbons (Kim et al., 2017). Therefore, the catalyst fraction in the CL must be tailored specifically to the reaction and the desired products.

Although GDEs have several advantages for CO_2_RR, they also face two significant challenges: flooding (Wang et al., 2019) and carbonation (Leonard et al., 2020). On the other hand, flooding is a problem for all GDEs, and it can cause severe hydrogen evolution in aqueous media. On the other hand, carbonation is unique to CO_2_RR, and it can compromise the stability and integrity of a GDE. GDE flooding is often associated with increasing current density and can lead to electrode failure. For instance, Leonard et al. (2020) found that a silver GDE remained stable for 5 h when the gas product had a constant CO fraction. However, when the current density exceeded 100 mA/cm_2_, a catastrophic failure occurred, and within 30 min, the GDE switched from producing CO to producing hydrogen. The double-layer capacitance at different current densities was linked to GDE flooding. This capacitance can predict flooding and GDE failure during CO_2_RR because an increase in capacitance signifies the expansion of the electrode–electrolyte interface into the GDE. Carbonation is a significant issue for GDEs, especially at higher current densities, because it can form crystallized salts and defects that allow water to percolate through the pores (Duarte et al., 2019). Overall, GDEs have a charge threshold for flooding, and failure begins with carbonate salt precipitation followed by electrolyte percolation into the crystals and GDE pores.

## 8. Scaling up and techno-economic evaluation of MES development for CO_2_RR

The research spectrum to develop MES or any technology can be divided into three stages: research, development, and deployment. Many studies are still in the fundamental research and development stage; thus, the true scope of deployment capabilities and business plans is yet to be realized in real-life scenarios except in theory. Until now, many studies related to scale-up MES were scarce as the technology is still focused on biocathode development; this is true as it is the major bottleneck in implementing MES. However, a development of large lab-scale operations of MES to produce hydrogen peroxide (H_2_O_2_) was done to study the ability of larger-scale MES. A 20-L double chamber MES was set up using graphite felt as electrodes. The results suggested that the production of H_2_O_2_ can be done with a rate of 10.82 mg L/h with an accumulation concentration of 454.44 mg/L within 42 h of operation at a stable cathode potential of −0.6 V with a high current density output of around 2.86 A/m^2^ (Zou et al., 2021) and this highlights the cornerstone in the MES development stage. However, the experiment only used the bioanode to dissociate water molecules into protons and electrons, simulating the microbial fuel cell (MFC) system. The absence of a biocathode in the system demonstrates the H_2_O_2_ as the product of the system. Thus, another biocathode research could significantly support the idea of large-scale MES.

Cost feasibility plays the most significant role in realizing CO_2_ sequestration when considering the scale-up pilot plant of MES. Most related studies on scale-up pilot plants reported that the highest proportionate cost to scale up the MES or any BES system mainly concerns the electrode cost because the material primarily consists of a noble metal such as platinum in the anode. Thus, it is one of the primary reasons why the study in MES focused on the fabrication of noble metal-free catalysts to reduce the cost at a competitive level as the other conventional methods. Together, these two components can reach up to 75% of the total cost of constructing a pilot-scale plant (Aiken et al., 2019). The summary of the expenses is illustrated in the pie chart below, which consists of all the major components required to build a pilot plant. However, this is true when factoring in the electrodes are made of noble metal, requiring massive capital to acquire. This problem can be solved by applying other BES fundamental research systems, specifically MFC that heavily relies on anode development, free from expensive noble metals. Through the synergetic approach with different systems, it is expected that the cost of the material could be substantially reduced.

**Figure 8:**
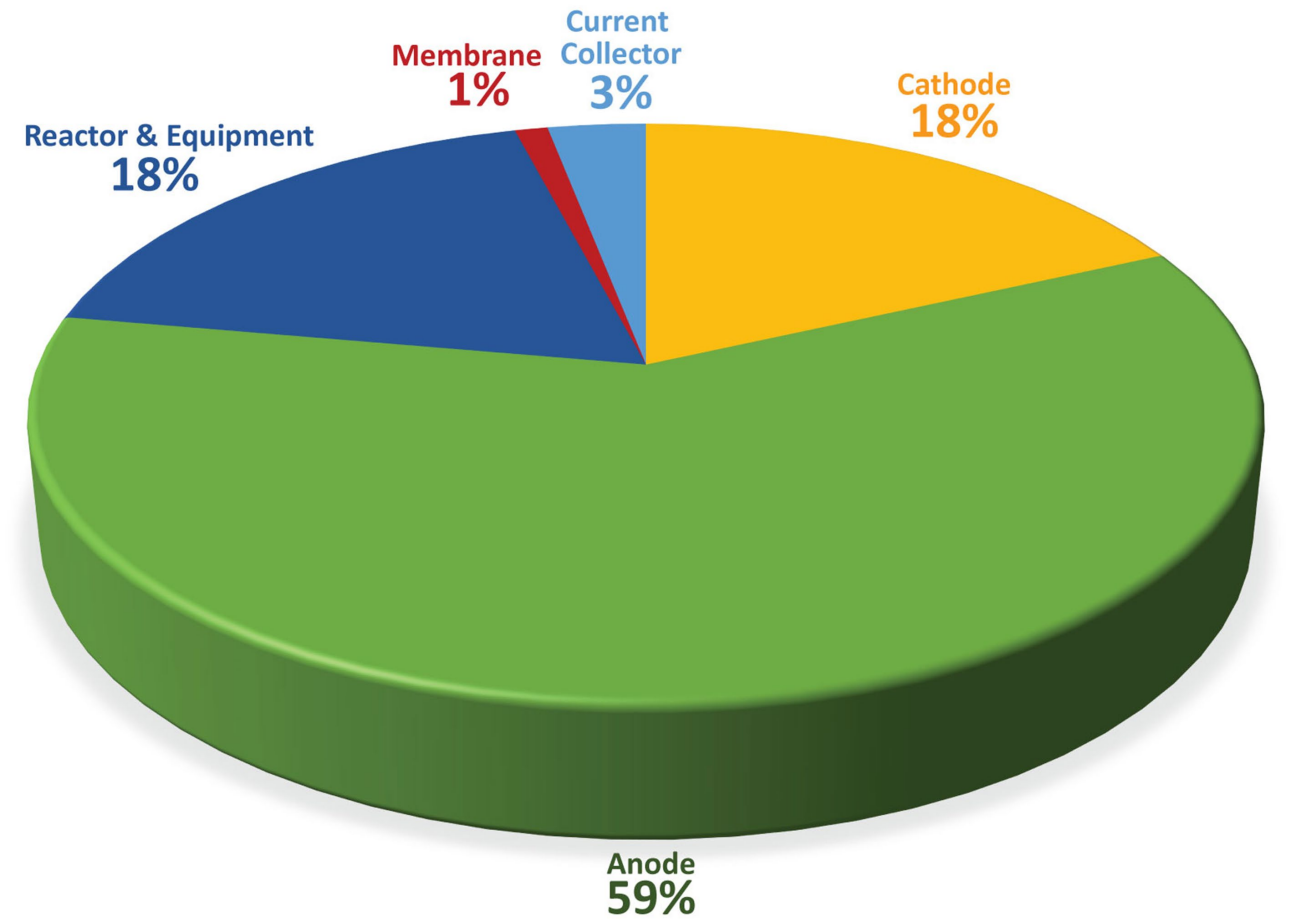
CAPEX estimation of MES components.

Regarding MES’s business development, two aspects frequently considered in setting up this technology mainly revolve around capital expenditure (CAPEX) and operating expenditure (OPEX). Most of the cost during the initial stage involves CAPEX. CAPEX covers the cost of equipment of the pilot reactor, such as electrode, membrane, reactor, current collector, gas tank, and piping (Figure 8). Meanwhile, OPEX covers most utilities, labor, maintenance, and raw materials production costs. Electricity plays a vital role in operating MES systems and can take up to 2/3 of the total cost of operation (Jourdin et al., 2020). Thus, specific measures could be installed by installing solar energy sources to reduce OPEX. Although this approach could incur high capital costs, the cost reduction could be felt in the long-term lifecycle of the plant.

Furthermore, applying MES as a standalone facility is currently not feasible due to its extended return of investment (ROI), and its restriction in current technology hinders the utilization of this biotic system on a large and industrial scale compared to an electrolyzer. However, several approaches that would be practical in the current state have been tested on a laboratory scale. Integrating MES technology in CO_2_ emitter facilities is promising because of the same area’s carbon substrate and biotransformation system source. The idea is tested using the CO_2_ from the brewery in synthesizing acetate, and the performance measure was the same as that of a pure carbon source (Roy et al., 2021), thereby proving the validity of the theory of integrating MES. Another interesting approach is to design MES in fossil fuel processing facilities that produce hydrogen and methane through steam reforming. This idea could be used to drive hydrogen-mediated MES while reducing the production of waste syngas.

Another method of integrating MES is coupling it with an anaerobic digester (AD), which does these two-system work in synergy. This approach uses the CO_2_ emitted from the anaerobic digestion of an organic matter, which is collected and supplied to MES to produce chemicals depending on the system’s configuration, such as volatile fatty acids or biomethane production, to increase AD output. This will reduce the waste generated while increasing the carbon recovery of the waste products by up to 98% (Aryal et al., 2020). In addition, coupling these two systems would incur a small fiscal burden as the system is not energy intensive in heat and electricity usage with minimum maintenance requirements. In terms of profitability, these secondary products that would be synthesized could offset the CAPEX and OPEX for a long time, thus making a surplus profit in future years. Another method of exploring industrial-size MES is by constructing it near green energy-producing facilities such as solar, wind and hydro. This could reduce the energy demand through energy transmission in on-site facilities.

## 9. Remaining challenges and future recommendation

In general, much work must be done in R&D regarding MES because it still needs to be feasible for real-world adaptations. Currently, most research addresses biocathode catalyst development, which is crucial in increasing the productivity of synthesized chemicals, high current density, and low overpotential. Although this news in MES development is good, as the cost heavily relies on the cathode catalyst, the high cost also includes the anode electrode, which most research should have focused on. This is important because the highly efficient anode could offset the energy requirement of the biocathode for CO_2_ reduction in terms of current density and oxygen evolution reaction (OER). The use of 3D printers is also an exciting insight worth exploring to build faster and more economical biocathode, which could lead to more discoveries in the future. Also, several other critical outlooks should be explored, such as:

- The use bioanode as a complementary electrode and biocathode. Using the MFC principle by coupling with MES would allow the MFC-MES system to work independently and minimize or eliminate the system’s need for external power. In addition, MFC could perform a secondary role in treating wastewater by using exoelectrogens to reduce COD and act as biosensors simultaneously. Thus, this idea could be used in exploring new research on integrating various BESs systems.
- Chain elongation-based MES should be explored more in producing a variety of compounds. Most MES focused on creating C1 and C2 compound, which is relatively easy to make, and the fundamental theory is known chiefly. However, the same could not be said for higher-chain combinations, as the metabolic pathways and process requirements are more complex and not fully realized.
- The biotic component of MES is another field of study that needs to be focused on. The exact mechanism of electron transfer and metabolic pathways of microbes could be exploited to modify microbes by increasing the CO_2_ reduction process and removing unnecessary pathways involved genetically. Thus, the genetic engineering field of study in MES is another frontier of research that needs to be broadened. Enzymatic electrosynthesis is one of the examples of metabolic engineering that addresses this area of research, as previously mentioned. Thus, more related research could be explored in this study area to realize this green technology development.
- The operation of the MES system is also one of the critical factors; these include the pH, temperature, mode of operations, mass transport, substrate, voltage and hydraulic retention are included in the optimization of MES. Some studies have shown that bioproducts produced under the same material with different conditions produce vastly different results.
- Lastly, pilot plants and scale-up are critical studies that must be addressed and realized to enable this technology to enter real-world applications. This is because the difference between bench-scaled MES against a more elaborated design construction where electrodes are stacked in intricate positions is more challenging to formulate and cannot be estimated through proportionate calculation and cost.

## 10. Conclusion

The MES is a BES technology proven to be a new study area with unrealized potential in combating climate change and increasing circular bioeconomy. The catalyst development of MES proved that many works in increasing the effectiveness of CO_2_ reduction via microbes were done previously. This review also covers the metabolic pathways related to the process, which could help harness it to improve the process, such as hydrogen-mediated pathways. Many approaches are being applied in modifying the biocathode through different ranges, from conventional methods to new methods, materials, and technology strategies, to transform CO_2_ into various chemicals. All new research mainly focused on a noble free metal catalyst that could be fabricated economically and work better than previous studies to achieve the final result, the biocompatible electrode. Also, some new key finding, such as 3D printer is an interesting method of biocathode preparation and is a promising technology for making better biocathodes. The factors of the MES process are also discussed to optimize the process better and with the idea of scaling up the MES for deployment on an industrial scale. All the key findings in MES research could help achieve CCU and decarbonise the economy, thus following the global aspiration to pursue sustainable development goals.

## Author contributions

II, KB, and MH prepared and wrote the manuscript. MS carried out the supervision with support from SL, MA, KL, and BJ. All authors contributed to the article and approved the submitted version.

## Conflict of interest

The authors declare that the research was conducted in the absence of any commercial or financial relationships that could be construed as a potential conflict of interest.

## Publisher’s note

All claims expressed in this article are solely those of the authors and do not necessarily represent those of their affiliated organizations, or those of the publisher, the editors and the reviewers. Any product that may be evaluated in this article, or claim that may be made by its manufacturer, is not guaranteed or endorsed by the publisher.
